# ABCG2: A Milestone Charge Model for Accurate Solvation Free Energy Calculation

**DOI:** 10.1021/acs.jctc.5c00038

**Published:** 2025-03-11

**Authors:** Xibing He, Viet H. Man, Wei Yang, Tai-Sung Lee, Junmei Wang

**Affiliations:** † Department of Pharmaceutical Sciences and Computational Chemical Genomics Screening Center, School of Pharmacy, University of Pittsburgh, Pittsburgh, Pennsylvania 15261, United States; ‡ Department of Chemistry and Biochemistry and Institute of Molecular Biophysics, Florida State University, Tallahassee, Florida 32306, United States; § Laboratory for Biomolecular Simulation Research, Center for Integrative Proteomics Research, and Department of Chemistry and Chemical Biology, Rutgers University, Piscataway, New Jersey 08854, United States

## Abstract

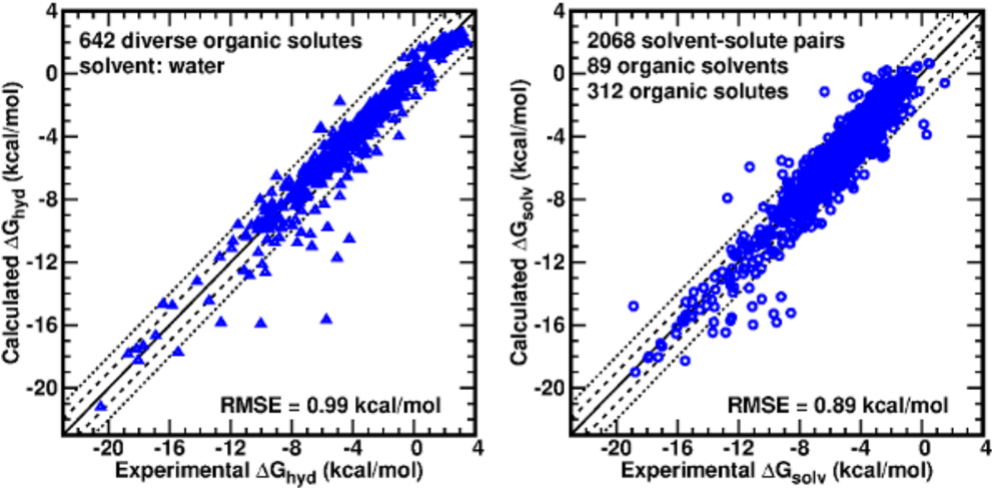

In this report, we describe the development and validation of ABCG2, a new charge model with milestone free energy accuracy, while allowing instantaneous atomic charge assignment for arbitrary organic molecules. In combination with the second-generation general AMBER force field (GAFF2), ABCG2 led to a root-mean-square error (RMSE) of 0.99 kcal/mol on the hydration free energy calculation of all 642 solutes in the FreeSolv database, for the first time meeting the chemical accuracy threshold through physics-based molecular simulation against the golden-standard data set. Against the Minnesota Solvation Database, the solvation free energy calculation on 2068 pairs of a range of organic solutes in diverse solvents led to an RMSE of 0.89 kcal/mol. The 1913 data points of transfer free energies from the aqueous solution to organic solvents obtained an RMSE of 0.85 kcal/mol, corresponding to 0.63 log units for logP. The benchmark on densities of neat liquids for 1839 organic molecules and heat of vaporizations of 874 organic liquids achieved a comparable performance with the default restrained electrostatic potential (RESP) charge method of GAFF2. The fluctuations of assigned partial atomic charges over different input conformations from ABCG2 are demonstrated to be much smaller than those of RESP from statistics of 96 real drug molecules. The validation results demonstrated not only the accuracy but also the transferability and generality of the GAFF2/ABCG2 combination.

## Introduction

Computer-aided drug design can significantly reduce the cost and time required for experimental trial and error through iterative compound synthesis and measurement. Computational protocols with even an accuracy level of 1–2 kcal/mol of root-mean-square error (RMSE) can be meaningful for active compound enrichment during early-stage ligand screening.^1^ However, in order to enable reliable ranking of candidate compounds, as required for the lead identification and optimization stages, the chemical accuracy level, as defined by the threshold of 1 kcal/mol of RMSE, must be reached.^2,3^

To achieve chemical accuracy, a variety of theoretical methods and practical computational tools have been developed, implemented, and even applied in drug discovery processes. Notably, to objectively assess these protocols on receptor–ligand binding prediction accuracy, several series of blind prediction challenges were organized in the past decades, such as the Drug Design Data Resource (D3R) (https://drugdesigndata.org) and the Statistical Assessment of the Modeling of Proteins and Ligands (SAMPL) (http://www.samplchallenges.org). Unfortunately, there have been no submissions close to the threshold of 1 kcal/mol.^4−8^ With the goal for drug discovery, the SAMPL series also contain challenges on host–guest binding affinities, distribution or partition coefficients of druglike molecules in or between immiscible solvents, and solvation free energies of organic compounds in water (specially referred as hydration free energies) or organic solvents.^9−12^ The rationale behind these challenges is that hydration/solvation, partition/distribution, host–guest binding, and protein–ligand binding are all transfer processes, i.e., transferring an organic molecule from one chemical environment to another chemical environment.^13^ During a transfer process, both the solute molecule and the solvent environment may need to rearrange accordingly.^13^ Therefore, the simple hydration/solvation process can serve as the baseline validation target for the assessment of molecular mechanics (MM) force fields (FFs), one of the two determining factors for molecular simulation [Monte Carlo (MC) or molecular dynamics (MD) simulation]-based free energy calculation quality.^14^ Improvement on hydration/solvation free energy prediction can be eventually translated to that on more complex processes, for instance, protein–ligand binding. In terms of hydration/solvation free energy prediction, a milestone goal of the entire topic has been to realize chemical accuracy prediction against the golden-standard FreeSolv data set,^15^ which contains 642 molecules (see the Supporting Information). As shown in Table 1, despite various efforts in FF-based molecular simulations,^16−28^ the quantum mechanical (QM) method combined with the polarizable continuum models,^29−31^ and machine learning (ML) methods,^32−34^ this milestone goal has not been previously reached. Note that in recent years, the ML and deep learning (DL) techniques have been applied to FF development (including charge model development). For instance, Mudedla et al. trained an ML model on atomic charges generated by density functional theory (DFT) for 31770 small molecules, but the model was only assessed by hydration free energy calculations for a small set of 33 solutes.^35^ Wang et al. applied a hybrid physical/graph neural network method to efficiently assign atomic charges mimicking the semiempirical quantum chemical methods AM1-BCC,^36^ and the performance of the model on hydration free energy calculation is close to that of AM1-BCC (Table 1).

**Table 1:** Benchmark Performance of Various Physics-Based Theoretical Methods or Machine Learning Methods on the Hydration Free Energy Calculation^a^^,^^b^

year	data	source	method/FF/charge model	MSE	MUE	RMSE
2009	504	Mobley et al.^16^	BAR/GAFF/AM1-BCC		0.68	1.26
2012	239	Shivakumar et al.^17^	FEP/OPLS2.0/CM1A-BCC		0.7	
2014	214	Koziara et al.^18^	TI/GROMOS53A6-UA or GROMOS-AA/ATB2.0	0.29, −0.90	1.61, 1.72	2.27, 2.47
2017	642	Matos et al.^19^	MBAR/GAFF/AM1-BCC	0.32	1.11	1.53
2017	426	Dodda et al.^20^	FEP/OPLS-AA/1.20*CM5, 1.14*CM1A or 1.14*CM1A-LBCC		0.61–1.26	
2018	426	Boulanger et al.^21^	FEP/GAFF-LJ*/GAAMP		0.79	
2018	613	Riquelme et al.^22^	MBAR/GAFF/polarized Hirshfeld-I or MBIS		1.53, 2.39	2.02, 2.92
2018	685	Stroet et al.^23^	TI/GROMOS 54A7/ATB3.0		0.91	1.32
2021	418	Lu et al.^24^	FEP/OPLS3e	0.50		1.02
2023	86	Boothroyd et al.^25^	MBAR/OpenFF2.0.0 “Sage”	0.44		1.09
2023	621	Karwounopoulos et al.^26^	Transformato/CGenFF v4.6		1.12	1.76
2024	623	Chakravorty et al.^27^	MBAR/CGenFF		1.18	2.04
2024	589	Karwounopoulo et al.^28^	Openmmtools/OpenFF2.0		1.01	1.33
2020	619	Luukkonen et al.^29^	molecular DFT	0.07	1.07	1.49
2023	642	Casillas et al.^30^	3D-RISM with various corrections	–0.21–0.00	1.01–1.32	1.44–1.80
2023	642	Stahn et al.^31^	polarizable continuum model CPCM-X		1.38	1.88
2023	642	Wee et al.^32^	various ML methods and the persistent Dirac models			1.64–2.11
2023	642	Xiang et al.^33^	Gaussian process regression using a marginalized graph kernel (GPR-MGK)			2.75
2023	642	Xu et al.^34^	various ML methods			1.21–3.35
2024	642	Wang et al.^36^	graph neural network			1.72

a The unit of MSE, MUE, and RMSE is kcal/mol.

b MSE, mean signed error; MUE, mean unsigned error; RMSE, root-mean-square error; BAR, Bennett acceptance ratio; MBAR, multistate Bennett acceptance ratio; FEP, free energy perturbation; TI, thermodynamic integration; ML, machine learning; and DFT, density functional theory. Other abbreviations are special names for different force fields, charge models, or various methods.

As one of the pioneer general FFs for arbitrary organic druglike molecules, the general AMBER force field (GAFF)^37,38^ and its current second generation (GAFF2), both of which were developed by our team, have been widely applied by researchers in a variety of research areas (Supporting Information, Figure S1). In practice, users of GAFF and GAFF2 often adopt the Austin Model 1 with the bond charge correction (AM1-BCC) charge model^39,40^ instead of the default restrained electrostatic potential (RESP) charge model^41,42^ for partial atomic charge generation because (1) AM1-BCC charge generation is very efficient (with no need of QM geometry optimization and calculation of the electrostatic potential on the input molecule) and convenient (with the help of the Antechamber module^38^ in the AmberTools) and (2) generated atom charges are less dependent on input conformations (see the articles on the AM1-BCC model by Jakalian et al.^39,40^ and the Discussion section). Despite its early occurrence compared to other general FFs or models, the combination of GAFF and AM1-BCC (GAFF/AM1-BCC) is still among the top performance list on hydration free energy benchmarking, as shown in Table 1. Recently, we developed a new set of BCC parameters and named the corresponding new charge model as ABCG2 (an abbreviation of AM1-BCC-GAFF2).^13^ The combination of GAFF2 parameters and ABCG2 atomic charges (GAFF2/ABCG2) significantly improved the calculation accuracy on hydration free energies (Δ*G*_hyd_) of 442 neutral molecules with diverse functional groups and solvation free energies (Δ*G*_solv_) of 895 pairs of diverse organic solvent–solute systems.^13^

In this manuscript, we first report our effort in expanding the ABCG2 charge model into a broader chemical space and structurally more complicated organic molecules, which cover all of the 642 solutes in the FreeSolv database, and for the first time achieving the milestone goal with a mean signed error (MSE) of −0.10 kcal/mol, a mean unsigned error (MUE) of 0.57 kcal/mol, and an RMSE of 0.99 kcal/mol (Table 2). Furthermore, the derived charge model was directly applied to large scales of calculations of solvation free energies in organic solvents and the transfer free energies of solutes between organic solvents and water. Note that the assigned atomic charges are the same for a molecule, regardless of whether it serves as a solute or as a solvent, i.e, no special “tweaks” are adopted for organic solvents. The displayed excellent accuracy and transferability of the GAFF2/ABCG2 model among water and various polar and nonpolar organic solvents lay a solid foundation for prediction of other important properties of essentially similar processes, such as the membrane permeabilities, receptor–ligand binding affinities, and ADMET (absorption, distribution, metabolism, excretion, and toxicity) properties of molecules in drug design projects.

**Table 2:** Performance of Hydration Free Energy Calculation with Different Protocols on 2 Subsets or All Sets of the FreeSolv Database and on the ATB3.0 Water Validation Set^a^

protocols/sets	N data	MSE	MUE	RMSE	PI	R
Matos_GAFF_AM1-BCC_FreeSolv_p1^b^^,^^e^	441	0.58	0.89	1.11	0.94	0.95
Matos_GAFF_AM1-BCC_FreeSolv_p2^c^^,^^e^	201	–0.26	1.61	2.21	0.92	0.89
Matos_GAFF_AM1-BCC_FreeSolv_all^d^^,^^e^	642	0.32	1.14	1.54	0.95	0.93
GAFF2_AM1-BCC_FreeSolv_p1^f^	441	0.60	1.01	1.28	0.91	0.92
GAFF2_AM1-BCC_FreeSolv_p2^f^	201	0.04	1.71	2.42	0.91	0.86
GAFF2_AM1-BCC_FreeSolv_all^f^	642	0.42	1.22	1.71	0.93	0.91
GAFF2_ABCG2_FreeSolv_p1^g^	441	–0.00	0.37	0.50	0.99	0.98
GAFF2_ABCG2_FreeSolv_p2^g^	201	–0.32	1.02	1.60	0.94	0.94
GAFF2_ABCG2_FreeSolv_all^g^	642	–0.10	0.57	0.99	0.98	0.97
GAFF2_RESP_FreeSolv_p1^h^	441	0.39	0.91	1.25	0.91	0.91
GAFF2_RESP_FreeSolv_p2^h^	201	0.43	1.39	1.90	0.92	0.91
GAFF2_RESP_FreeSolv_all^h^	642	0.40	1.06	1.48	0.93	0.93
GAFF2_ABCG2_ATB3.0WaterValidation^g^^,^^i^	685	–0.05	0.52	0.79	0.98	0.98

a The unit of MSE, MUE, and RMSE is kcal/mol. Raw data are shown in the spreadsheet file in the Supporting Information.

b Subset FreeSolv_p1 contains 441 molecules, which were covered in ref (13).

c Subset FreeSolv_p2 contains the remaining 201 molecules in FreeSolv but not in FreeSolv_p1.

d FreeSolv_all contains all 642 molecules in FreeSolv.

e Statistical data here were analyzed from the original raw calculation data in ref (19), which were calculated with the GAFF/AM1-BCC combination.

f Calculated with the GAFF2/AM1-BCC combination.

g Calculated with the GAFF2/ABCG2 combination.

h Calculated with the GAFF2/RESP combination.

i The ATB3.0 water validation set from ref (23).

## Computational Methods

### Preparation of Benchmarked Data Sets

The experimental Δ*G*_hyd_ data were taken from the FreeSolv v0.52 database^15^ (https://github.com/MobleyLab/FreeSolv) and the ATB3.0 water validation set^23^ (https://github.com/ATB-UQ/ATB3.0_validation_data). The experimental Δ*G*_solv_ data in organic solvents were obtained from the Minnesota solvation (MNSol) database v2012 (https://license.umn.edu/product/minnesota-solvation-mnsol-database).^43^ The initial structures of solutes/solvents were taken from the mol2 files in FreeSolv v0.52 or from the pdb files in the ATB3.0 water validation set or from the xyz files in MNSolv. These structure files were imported to Schrödinger Maestro v11.2 (Schrödinger, LLC, New York, NY) for visual check and necessary manual modifications, such as setting correct bond types for the structures in the pdb or xyz files, and then all of the structures were saved in the mol2 format.

### Generation of Atomic Charges and Force Field Parameters

The mol2 files of molecules were fed to the modified Antechamber^38^ module in AmberTools,^44^ which calls the Sqm module to perform geometry optimization and generate a topology file of optimized conformation. An option of “-c abcg2” was added to the modified Antechamber module to assign atomic charges utilizing the ABCG2 charge model, just as the “-c bcc” option which had been already in Antechamber to assign AM1-BCC charges (see the table of “List of the Charge Methods” on pages 312–313 in the Amber 2024 Reference Manual). In order to assign the RESP charges, the mol2 files of molecules were modified and fed to Gaussain16^45^ to conduct geometry optimization and single-point calculation at the level of HF/6-31G*, and then the Gaussian output files were fed to Antechamber to generate RESP partial charges with the option of “-c resp”. The Parmchk2 module (with the option of “-s 2”) in AmberTools was used to generate GAFF2 bonded and van der Waals parameters. The TIP3P water model^46^ was adopted to handle explicit water molecules since it is the default water model for GAFF, GAFF2, and AMBER protein force fields from ff99SB to ff14SB.

### Preparation of Simulated Systems

For each solvent–solute pair, a gas-phase system of the solute only and a liquid system of solvating a single solute molecule in a cubic box of pure solvent molecules were generated. The solvation process was carried out with the tleap module in AmberTools. The number of the solvent molecules varies for each system so that the final equilibrated cubic simulation cell has a box side of approximately 40 Å, which can guarantee a minimum distance of 10 Å (the short-range cutoff distance) from any solute atom to the edges of the cubic simulation box to avoid image violation. Pre-equilibrated (5 ns) bulk solvents were prepared before the solutes were solvated in organic solvents.

### MD Simulations and Alchemical Free Energy Calculations

Alchemical free energy simulations were performed in both the solvated and gas phases to determine solvation free energy differences. The alchemical enhanced sampling (ACES) method^47^ implemented in the GPU-accelerated AMBER22 program^48^ was employed for these calculations. ACES conducted thermodynamics integration (TI) through a Hamiltonian replica exchange MD (HREMD) framework using a new design of alchemical transformation pathways^49^ and smooth step softcore potentials.^47^

Eleven alchemical states (λ = 0.0, 0.1, 0.2, 0.3, 0.4, 0.5, 0.6, 0.7, 0.8, 0.9, and 1.0) were used for both the solvated and gas phases. Four independent runs were carried out for each system (gas and solvated). The final free energy results were obtained by subtracting the gas-state result from the solvated-state result.

A 1 fs time step was used. Each λ state underwent geometry minimization to avoid steric crashes, followed by equilibration. The gas system was equilibrated for 0.5 ns in the NVT ensemble at 298 K. The liquid system was first equilibrated for 0.5 ns in the NVT ensemble and then for 3.0 ns in the NPT ensemble at 1 atm and 298 K. HREMD simulations were then conducted for 2.0 ns with 100,000 Hamiltonian replica exchange attempts and 20 MD steps between each attempt, resulting in an equivalent of 2,000,000 MD steps for each λ state. A Monte Carlo barostat^50^ and a Langevin thermostat^51^ were used to maintain a pressure of 1 atm and a temperature of 298 K.

### Transfer Free Energy and log *P*

The transfer free energy Δ*G*_trans_ of a solute from solvent a to solvent b is calculated from the solvation free energies of the solute in solvent a (Δ*G*_solv,a_) and solvent b (Δ*G*_solv,b_)
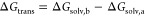
1and the corresponding log *P* is calculated by
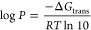
2

## Results

### Performance on Δ*G*_hyd_ Calculation on the FreeSolv v0.52 Data Set

We optimized the bond charge correction (BCC) parameters in our ABCG2 charge model, targeting the experimental Δ*G*_hyd_ data of representative molecules with various functional groups, and conducted verification on the whole FreeSolv database version 0.52 (FreeSolv v0.52).^15^ This database covers a diverse chemical space of organic molecules (containing H, C, N, O, S, P, F, Cl, Br, and I atoms) and a broad range of experimental Δ*G*_hyd_ data (from −25.5 to 3.4 kcal/mol). The molecular weight (MW) of the solutes ranges from ∼16 to ∼499 g/mol (Supporting Information, Figure S2), which are all less than 500 g/mol and fit the typical sizes of druglike molecules according to Lipinski’s “rule of five”.^52^ Furthermore, most of the solutes have a MW less than 300 g/mol (Supporting Information, Figure S2), falling in the range of leadlike molecules based on Congreve’s “rule of three”.^53^

Our first version of the ABCG2 charge model^13^ was verified on 441 out of 642 solutes in the FreeSolv v0.52 data set (referred to here as FreeSolv_p1 in Table 2), which are mostly monofunctional molecules. The remaining 201 molecules (referred to here as FreeSolv_p2, as listed in Table 2) are usually larger polyfunctional molecules or contain phosphorus (P) atoms, which were not verified or not covered in our first version of the ABCG2 charge model. After further optimization and expansion of BCC parameters, our current (second) version of the ABCG2 charge model covers all involved bond types in the 642 molecules of the whole FreeSolv v0.52 data set (referred to here as FreeSolv_all, as listed in Table 2). Table 2 lists the statistical analysis of the metrics including the mean of signed error (MSE), mean of unsigned error (MUE), RMSE, predictive index (PI),^54,55^ and Pearson’s correlation coefficient (R) on Δ*G*_hyd_ calculation of the FreeSolv data set with the combinations of GAFF2/AM1-BCC or GAFF2/ABCG2 (raw data in the spreadsheet file in the Supporting Information). It is clear that compared to GAFF2/AM1-BCC, GAFF2/ABCG2 significantly reduced the MUE from 1.22 to only 0.57 kcal/mol for all 642 solutes and reduced the RMSE from 1.71 to 0.99 kcal/mol, breaking the threshold of RMSE = 1 kcal/mol. The PI increased from 0.93 to 0.98, and the R increased from 0.91 to 0.97.

**Figure 1:**
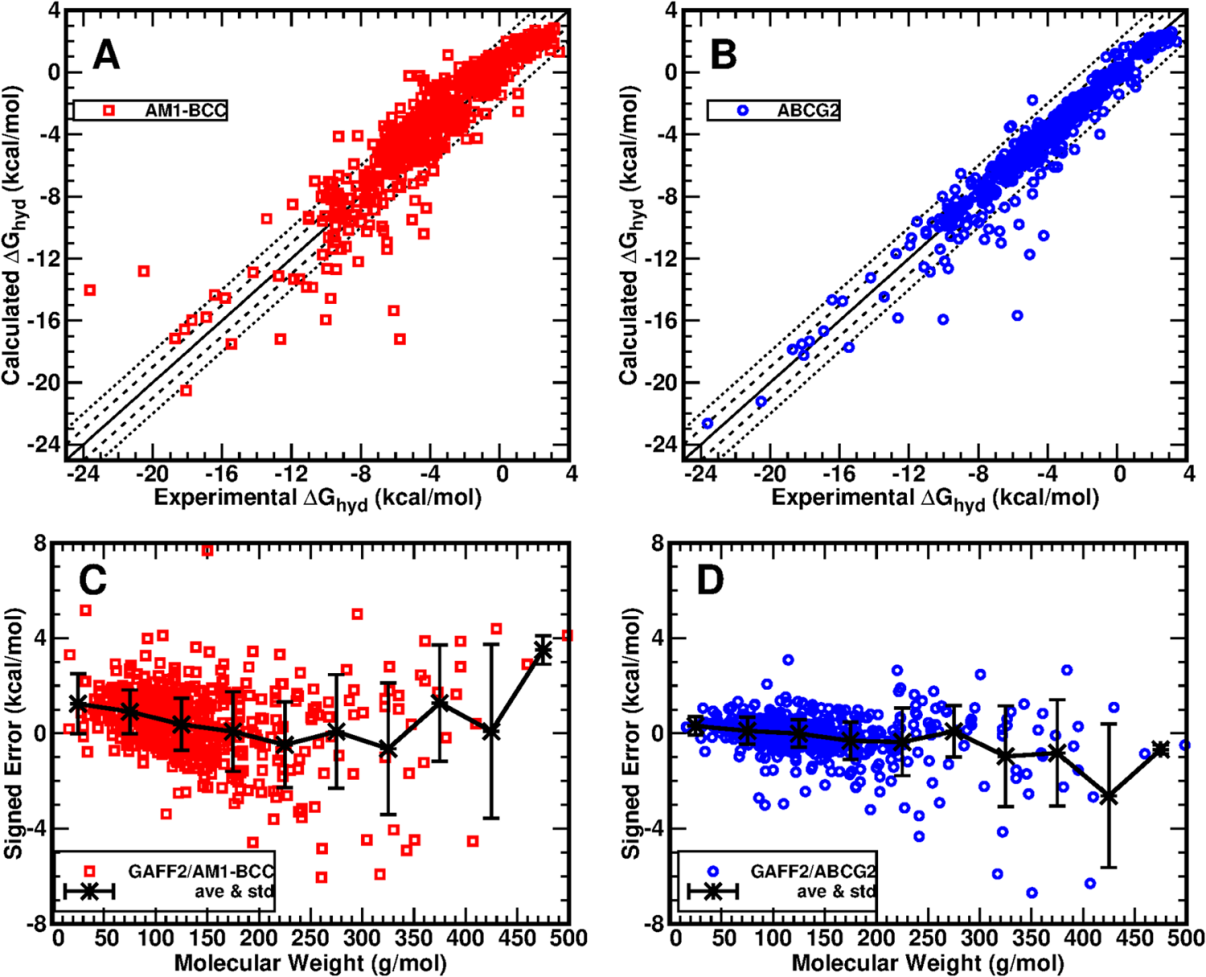
Performance of hydration free energy (Δ*G*_hyd_) calculation on all 642 organic solutes in the FreeSolv data set using GAFF2 force field parameters with either AM1-BCC or ABCG2 charges. (A) Δ*G*_hyd_ calculated with GAFF2/AM1-BCC versus experimental values. (B) Δ*G*_hyd_ calculated with GAFF2/ABCG2 versus experimental values. (C) Distribution of signed errors from GAFF2/AM1-BCC along the molecular weight of solute molecules. (D) Distribution of signed errors from GAFF2/ABCG2 along the molecular weight of solute molecules.

Figure 1A,1B show the scatter plots of experimental Δ*G*_hyd_ versus calculated Δ*G*_hyd_ by applying GAFF2 combined with the AM1-BCC and ABCG2 charge models, respectively. Note that in our ACES protocol, 4 independent runs were carried out for both the gas and aqueous phases of a solute, and the averaged calculation values were used as the final result, and the standard deviations were reported in the “hydration” sheet in the Supporting Excel file. The standard deviations from the 4 independent runs are usually less than 0.2 or 0.3 kcal/mol, which are all much smaller than the default experimental uncertainty (0.6 kcal/mol) in the FreeSolv database. Based on this fact, we did not plot the error bars in Figure 1A,1B for the sake of clarity. Besides the overall narrower distribution of data points around the ideal distribution (the solid lines), the ABCG2 data points concentrate (86.4%) within the region of ±1 kcal/mol (the dashed lines) compared to AM1-BCC data points (51.2%). Also, there are significantly fewer outliers outside of the region of ±2 kcal/mol (the dotted lines) in the ABCG2 data points (4.7%) than the AM1-BCC data points (14.2%). A close check of these outlier molecules reveals that most of them contain sulfur (S) and phosphorus (P) atoms, and these molecules happen to have an experimental uncertainty greater than 1 kcal/mol according to the FreeSolv database. We sincerely expect experimental communities to conduct high-quality measurements to verify and obtain high-quality experimental values so that we can further optimize our charge model accordingly. But so far, such a larger experimental uncertainty plus the more complex chemical component and structure makes these molecules with S and P atoms typically difficult objects of Δ*G*_hyd_ calculation; therefore, they were often excluded by previous benchmark studies (Table 1); for example, they were explicitly mentioned in refs (23,26,28). Karwounopoulos et al. benchmarked CGenFF on Δ*G*_hyd_ calculation of 621 out of 642 molecules in the FreeSolv database (Table 1).^26^ They excluded 10 molecules, the parameters of which the cgenff program failed to generate, and also excluded the 11 organophosphorodithioates.^26^ Karwounopoulos et al. also benchmarked OpenFF2.0 recently on the Δ*G*_hyd_ calculation of 589 out of 642 molecules in FreeSolv, and the excluded compounds include all P-containing compounds.^28^ Our calculations in this study covered all 642 compounds in the FreeSolv database. The improvement of GAFF2/ABCG2 compared to GAFF2/AM1-BCC is also exhibited by the probability density distributions of errors (Figure S3A).

We also analyzed the raw calculation data with GAFF/AM1-BCC by Matos et al.,^19^ as exhibited in Table 2. It is noted that the statistical performances of GAFF/AM1-BCC and GAFF2/AM1-BCC are very close, with GAFF/AM1-BCC being slightly better than GAFF2/AM1-BCC. However, we would like to emphasize that this does not mean that GAFF is better than GAFF2. On the contrary, the overall performance of GAFF2 is generally better than that of GAFF, as shown by examples listed in ref (13). Both GAFF and GAFF2 were developed by us based on the default RESP charge model, just as other biomolecular FFs in the Amber family. In fact, the performance of GAFF2/RESP on Δ*G*_hyd_ calculations is better than those of both GAFF/AM1-BCC and GAFF2/AM1-BCC, as demonstrated by the corresponding MSE, MUE, and RMSE values in Table 2. We have always been encouraging users to adopt RESP charges with GAFF and GAFF2 when applied together with Amber protein FFs, unless otherwise clearly stated.^56^ However, we also noticed the overwhelming popularity of semiempirical charge models like AM1-BCC among the applications of GAFF (GAFF2) and were well aware of its aforementioned advantages; hence, we were inspired to optimize the BCC parameters targeting critical experimental properties like Δ*G*_hyd_/Δ*G*_solv_.

Table 2 reveals that the MSE jumped down by 0.84 kcal/mol from FreeSolv_p1 to FreeSolv_p2 for GAFF/AM1-BCC, a slightly smaller magnitude of 0.56 kcal/mol for GAFF2/AM1-BCC, but a much smaller magnitude of only 0.32 kcal/mol for GAFF2/ABCG2. Considering the general size difference between FreeSolv_p1 and FreeSolv_p2 subsets, such a different trend can be better exhibited as the averaged signed errors (SEs) as a function of the solutes’ MWs (Figure 1). Figure 1C clearly shows that as the solutes’ MW increases from 0 to 250 g/mol, the averaged SE decreases from ∼1.2 to ∼−0.5 kcal/mol for GAFF2/AM1-BCC. On the other hand, for GAFF2/ABCG2 (Figure 1D), the averaged SEs are almost flat around 0 in the same region of the MW. The averaged SEs fluctuate as the MW > 250 g/mol as the data points become much scarcer, indicating that more reliable experimental data in the heavy MW region are needed to verify the trend.

**Figure 2:**
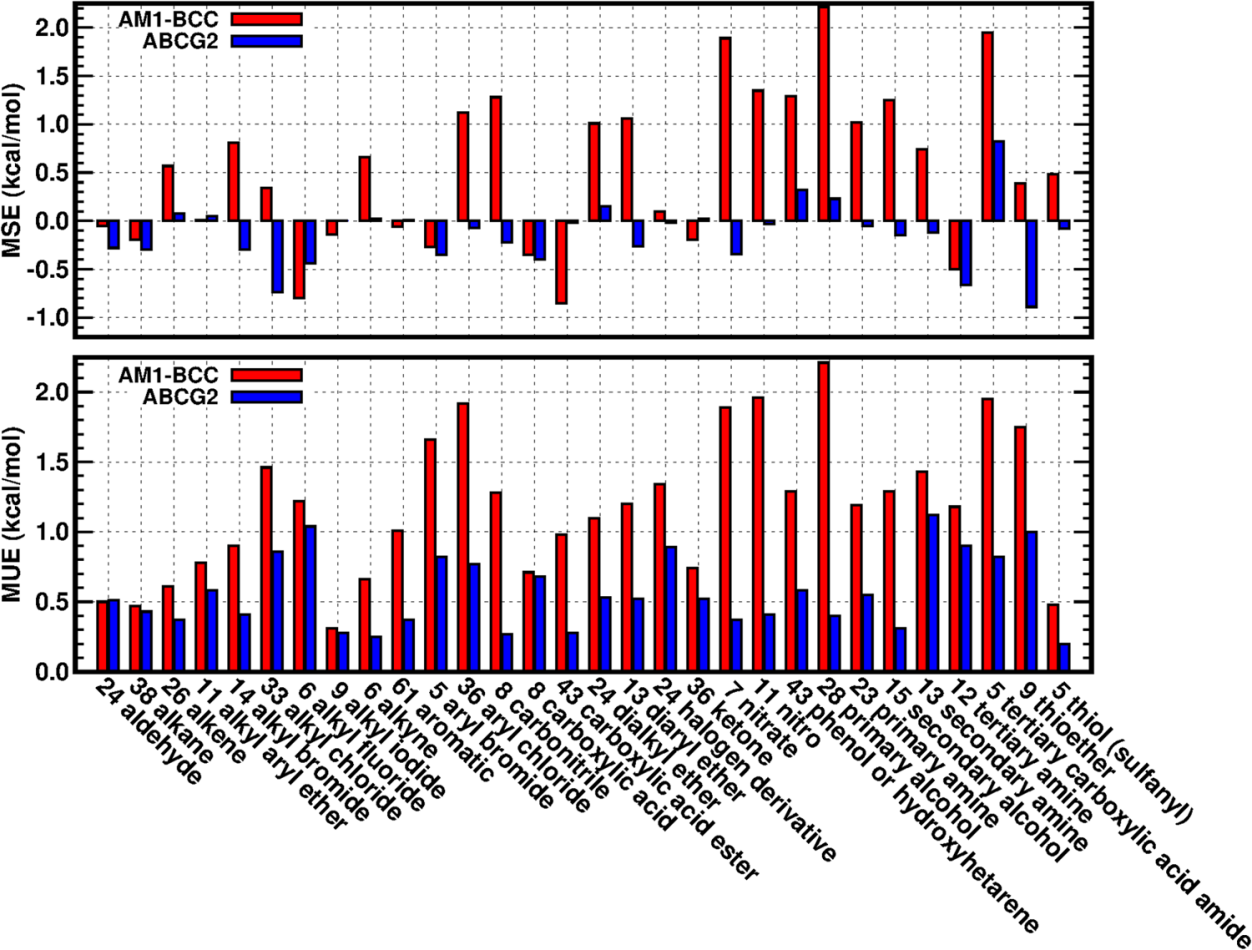
Performance of hydration free energy calculation with GAFF2/AM1-BCC (red bars) or GAFF2/ABCG2 (blue bars) on the functional groups of solutes in the FreeSolv data set. In the labels of the *X*-axis, the number before the group name is the number of solutes in the corresponding group.

Similar to the analysis carried out by Karwounopoulos et al.,^26^ we also categorized the 642 solutes based on their first chemical functionalities described in the groups.txt file from the FreeSolv repository. For the 30 functional groups that contain at least 5 solutes in FreeSolv, the MSEs and MUEs of solutes in each group from GAFF2/AM1-BCC or GAFF2/ABCG2 are calculated and shown in Figure 2. For all functional groups, the MUEs from GAFF2/ABCG2 (blue bars) are smaller than the MUEs from GAFF2/AM1-BCC (red bars). For half of the 30 functional groups, the MUEs from GAFF2/ABCG2 (blue bars) are less than 0.5 kcal/mol. There are only two functional groups (alkyl fluorides and secondary amines) whose MUEs from GAFF2/ABCG2 are slightly higher than 1 kcal/mol.

### Performance on Δ*G*_hyd_ Calculation on the ATB3.0 Water Validation Set

Stroet et al. collected 773 published experimental Δ*G*_hyd_ data (including those in FreeSolv) and then excluded 88 solutes (59 in FreeSolv and 29 not in FreeSolv) with an experimental uncertainty greater than 1 kcal/mol.^23^ The resulting set of 685 molecules (583 in FreeSolv and 102 not in FreeSolv) was “referred to as the ATB3.0 water validation set” by Stroet et al.^23^ We benchmarked GAFF2/ABCG2 on this ATB3.0 water validation set (raw data in the spreadsheet in the Supporting Information) and displayed the summarized results in Table 2. The magnitudes of the MUE (0.52 kcal/mol) and RMSE (0.79) kcal/mol are all smaller than those from the GROMOS 54A7 force field^57^ with ATB3.0 generated atom charges (MUE = 0.91 kcal/mol, RMSE = 1.32 kcal/mol, as shown in Table 1). Again, the RMSE of 0.79 kcal/mol from our GAFF2/ABCG2 protocol on this set of 685 solutes went beyond the threshold of RMSE = 1 kcal/mol and even lower than the RMSE of 0.99 kcal/mol on the 642 solutes of FreeSolv due to the exclusion of difficult molecules. As to the 102 solutes which are in the ATB3.0 data set but not in the FreeSolv data set, as shown in the spreadsheet file in the Supporting Information, GAFF2/ABCG2 leads to an MSE of −0.16 kcal/mol, an MUE of 0.74 kcal/mol, and an RMSE of 1.04 kcal/mol which is only slightly larger than the RMSE of 0.99 kcal/mol for the FreeSolv data set.

### Comparison to State-of-the-Art Polarizable Force Fields

Polarizable FFs can deal with the varied electrostatic effects across different dielectric environments^58−60^ and are supposed to produce molecular properties like Δ*G*_hyd_/Δ*G*_solv_ more accurately via molecular simulations. While being considered the next generation of FFs and under active development,^58−60^ polarizable FFs have not arrived at the main-steam status of fixed-charged additive FFs like GAFF/GAFF2,^13,37^ CGenFF,^61−63^ OPLS,^24^ GROMOS,^57^ etc. due to efficiency and comprehensiveness reasons. Indeed, the success from this work further strengthens our belief that the accuracy of additive FFs can be further improved. Very recently, Pereyaslavets et al. developed a multipolar polarizable FF (named ARROW FF), which was fitted entirely to ab initio calculations and which can be applied in Path Integral MD (PIMD) simulations incorporating quantum mechanics and hence including nuclear quantum effects.^64^ For a selected set of 34 solutes including various functional groups, PIMD with this polarizable ARROW FF produced an MUE of 0.22 kcal/mol on Δ*G*_hyd_ calculation, compared to an MUE of 0.88 kcal/mol from classical MD with fixed-charge GAFF/AM1-BCC, and an MUE of 0.76 kcal/mol from MD with another polarizable model AMOEBA.^64,65^ For comparison, classical MD with our GAFF2/ABCG2 model in this work achieved an MUE of 0.36 kcal/mol for the same set, better than the result from the polarizable AMOEBA model and close to the result from the polarizable ARROW FF applied in PIMD with nuclear quantum effects.

### Performance on Δ*G*_solv_ Calculation on the MNSol-v2012 Data Set

**Figure 3:**
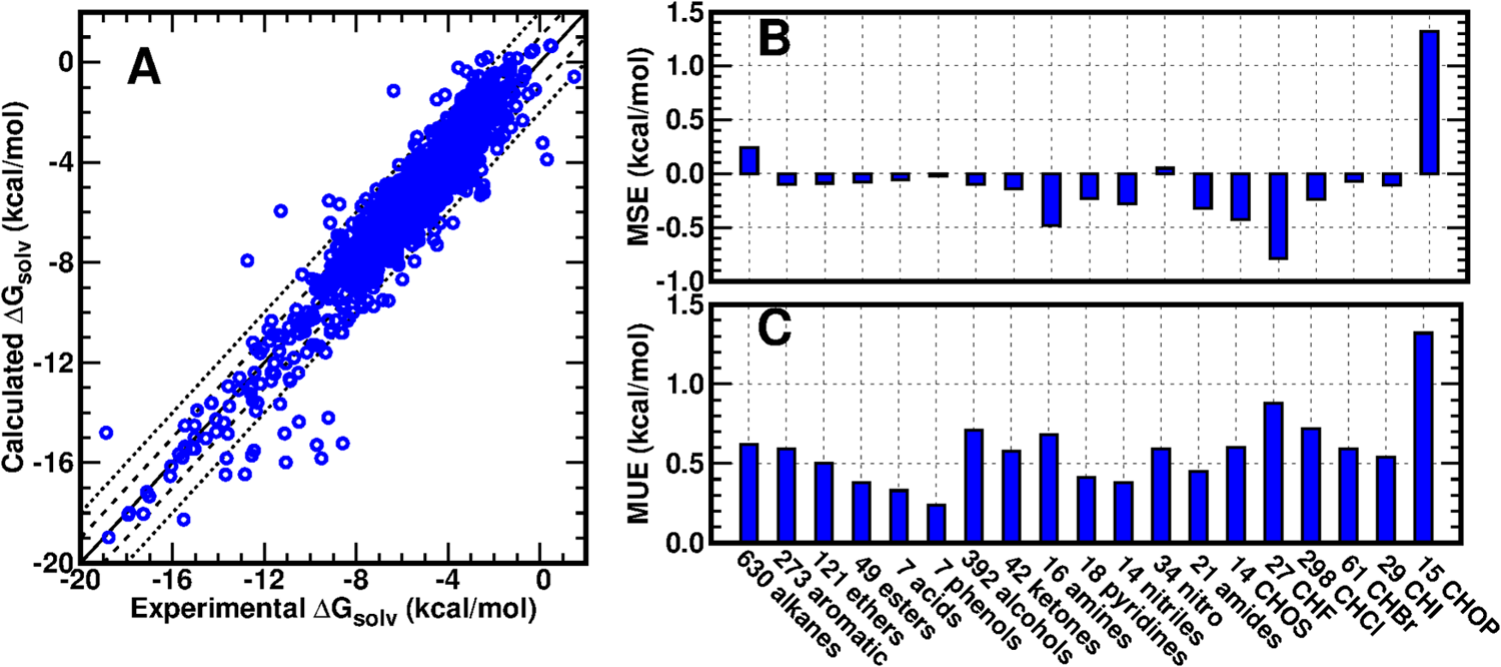
Performance of solvation free energy (Δ*G*_solv_) calculation on 2068 organic solvent–solute pairs in the MNSol data set using GAFF2 force field parameters with ABCG2 charges. (A) Δ*G*_solv_ calculated values versus experimental values. (B, C) Mean of signed errors (MSEs) and mean of unsigned errors (MUEs) based on classifications of organic solvents. In the shared *X*-axis labels of panels (B, C), the number before each group name is the number of data points in the corresponding group, and the capital letters indicate the containing elements.

After optimization and verification of BCC parameters in the newly developed ABCG2 charge model targeting Δ*G*_hyd_, we directly applied GAFF2/ABCG2 on Δ*G*_solv_ calculations of neutral organic solutes in neutral organic solvents taken from the Minnesota solvation database version 2012 (MNSol-v2012).^43^ This database consists of 3037 experimental Δ*G*_solv_ or Δ*G*_trans_ for 790 solutes in 92 solvents. After excluding data about Δ*G*_trans_, charged ions, water, and a few inorganic molecules, a data set of 2068 experimental Δ*G*_solv_ was obtained, which covers 312 neutral organic solutes and 89 neutral organic solvents with dielectric constants ranging from 1.8 to 181.6. Note that no specific “tweaks” are adopted for organic solvents in our charge model: a molecule is assigned the same set of partial charges, regardless of whether it serves as a solute or as a solvent. Figure 3 shows the calculated Δ*G*_solv_ values versus the corresponding experimental values of these 2068 solvent–solute pairs. 52.1% of the data points have unsigned errors (UEs) < 0.5 kcal/mol, 59.7% have UEs < 0.6 kcal/mol, 81.2% have UEs < 1.0 kcal/mol (within the two dashed lines shown in Figure 3A), 93.3% have UEs < 1.5 kcal/mol, and 97.1% have UEs < 2.0 kcal/mol (within the two dotted lines shown in Figure 3A). Only 2.9% of data points have UEs > 2.0 kcal/mol. We also categorized all 2068 data points based on the classifications of the solvents and calculated the corresponding MSEs and MUEs, as shown in Figure 3B,3C. All solvent groups have magnitudes of the MSE and MUE of Δ*G*_solv_ less than 1.0 kcal/mol except for the CHOP solvent group (containing elements C, H, O, and P). Overall, GAFF2/ABCG2 achieved an MSE of −0.02 kcal/mol, an MUE of 0.63 kcal, RMSE = 0.89 kcal/mol, PI = 0.94, and *R* = 0.94 on the 2068 organic solvent–solute Δ*G*_solv_ calculations in the MNSol database. Such an accuracy is close to the aforementioned Δ*G*_hyd_ benchmark and also breaks the threshold of RMSE = 1 kcal/mol. Considering the broad range of the dielectric constant of the solvents, the Δ*G*_solv_ result also exhibited a good transferability of GAFF2/ABCG2 among water and various polar and nonpolar organic solvents. Such accuracy and transferability show great promise in its application in the accurate prediction of the free energies of more complex interactions, such as protein–ligand binding.

### Performance on Δ*G*_trans_ Calculation on the MNSol-v2012 Data Set

Among the 2068 organic solvent–solute pairs (involving 89 solvents and 312 solutes) in the MNSol data set, 1913 pairs (involving 89 solvents and 253 solutes) contain solutes which also appear as the hydration solutes in the FreeSolv data set. Therefore, Δ*G*_trans_ of the transferring corresponding solute from the aqueous solution to the corresponding organic solvent can be derived by eq 1 given in the Computational Methods section. For all 1913 Δ*G*_trans_ data points, MSE = −0.10 kcal/mol, MUE = 0.63 kcal/mol, and RMSE = 0.85 kcal/mol (Table 3 and the spreadsheet file in the Supporting Information), which correspond to MSE = 0.08 log units, MUE = 0.46 log units, and RMSE = 0.63 log units for logP (eq 2 given in the Computational Methods section). Table 3 also lists the statistic results of Δ*G*_trans_ for 11 organic solvents with the top number of Δ*G*_trans_ data involved.

**Table 3:** Performance of the Transfer Free Energy Calculation with GAFF2/ABCG2

solvents	N data	MSE (kcal/mol)	MUE (kcal/mol)	RMSE (kcal/mol)	PI	*R*
all	1913	–0.10	0.63	0.85	0.96	0.95
octanol–water	201	–0.22	0.63	0.87	0.94	0.93
hexadecane–water	178	0.06	0.52	0.76	0.97	0.97
chloroform–water	94	–0.85	0.98	1.31	0.95	0.93
cyclohexane–water	85	0.08	0.53	0.74	0.98	0.97
carbon tetrachloride–water	72	0.18	0.66	0.90	0.97	0.97
benzene–water	67	–0.23	0.76	1.37	0.93	0.87
heptane–water	63	0.30	0.64	1.05	0.99	0.98
diethyl ether–water	63	–0.29	0.59	0.72	0.97	0.96
hexane–water	56	0.14	0.64	0.96	0.97	0.96
xylene–water	46	–0.02	0.59	0.73	0.96	0.96
toluene–water	46	–0.06	0.57	0.67	0.96	0.96

## Discussion

As mentioned previously, RESP is the default method of assigning atomic charges in GAFF and GAFF2. In a FF, the nonbonded parameters such as atomic charges and van der Waals (VDW) parameters must work consistently with each other and with the bonded parameters (bonds, valence angles, dihedral, and improper angles). They are usually developed and adjusted iteratively. Hence, it is natural for users to wonder how much the effects would be brought on other properties if ABCG2 charges are adopted instead of RESP charges: for instance, the bulk properties of pure liquids like density and heat of vaporization, the dipole moment of organic molecules in gas and in aqueous solution, and the free energy of biophysical processes.

As exhibited in Table 2, the Δ*G*_hyd_ calculations on the whole FreeSolve data set (642 solutes) by GAFF2/RESP lead to an MSE of 0.40 kcal/mol, an MUE of 1.06 kcal/mol, and an RMSE of 1.48 kcal/mol, which is very competitive and remains in top positions in comparison with other FFs on the Δ*G*_hyd_ benchmark, as listed in Table 1. GAFF2/AM1-BCC leads to slightly worse results on the same data set: MSE = 0.42 kcal/mol, MUE = 1.22 kcal/mol, and RMSE = 1.71 kcal/mol. GAFF2/ABCG2 significantly improves the performance of Δ*G*_hyd_ calculations on the same data set, which is the key point of this study: MSE is −0.10 kcal/mol, MUE is 0.57 kcal/mol, and RMSE is 0.99 kcal/mol.

We calculated the densities of neat liquids for 1839 organic molecules under 1.0 bar and the room temperatures corresponding to experimental measurements in both GAFF2/RESP and GAFF2/ABCG2. The experimental data were collected from the chapter of Physical Constants of Organic Compounds in the CRC Handbook of Chemistry and Physics (90th Edition).^66^ The calculated raw data are listed in the spreadsheet file in the Supporting Information. As a statistical summary of results, GAFF2/RESP resulted in an MSE of 0.010 g/cm^3^, an MUE of 0.022 g/cm^3^, and an RMSE of 0.036 g/cm^3^ (Table S1); if considering the percent errors, the mean signed percent error (MSPE) is 1.10%, the mean unsigned percent error (MUPE) is 2.21%, and the root mean squared percent error (RMSPE) is 3.54% (Table S1). The statistical results from GAFF2/ABCG2 are very close: MSE = 0.011 g/cm^3^, MUE = 0.023 g/cm^3^, RMSE = 0.037 g/cm^3^, MSPE = 1.19%, MUPE = 2.24%, and RMSPE = 3.56% (Table S1). Figure S4A,B in the Supporting Information show the scatter plots of density data points calculated by GAFF2/RESP or GAFF2/ABCG2 versus the experimental values; Figure S4C shows the scatter plot of density data points calculated by GAFF2/RESP versus the values calculated by GAFF2/ABCG2, which demonstrates a very high correlation: the mean signed deviation (MSD) is 0.001 g/cm^3^, the mean unsigned deviation (MUD) is 0.009 g/cm^3^, the root-mean-square deviation (RMSD) is 0.016 g/cm^3^, and *R* is 0.999. Figure S4D shows that the probability distributions of the SE for 1839 density data from GAFF2/RESP and GAFF2/ABCG2 are almost identical.

We also calculated the heats of vaporization (*H*_vap_) of 874 organic liquids (see the spreadsheet file in the Supporting Information) in GAFF2/RESP and GAFF2/ABCG2, respectively, and compared them to the experimental data.^67^ The results from GAFF2/RESP are as follows: MSE = −0.84 kcal/mol, MUE = 1.54 kcal/mol, RMSE = 1.88 kcal/mol, MSPE = −7.08%, MUPE = 14.08%, and RMSPE = 17.55% (Table S2). The results from GAFF2/ABCG2 are as follows: MSE = −0.67 kcal/mol, MUE = 1.38 kcal/mol, RMSE = 1.74 kcal/mol, MSPE = −5.30%, MUPE = 12.70%, and RMSPE = 16.20% (Table S2). The RMSPE may seem larger than previous small-scale benchmark studies on heats of vaporization by other general FFs,^61^ but we would like to point out that our benchmark study was conducted on 874 compounds, which is one order larger than the previous benchmark and probably the largest scale of benchmark on calculating heats of vaporization, as far as we know. The reliability of the experimental data for heats of vaporization is also weaker than other properties like density due to the large uncertainties from different resources.^67^ Here, we care more about the effects of changing the charge assignment method from RESP to ABCG2. We can see that the results for heats of vaporization from GAFF2/ABCG2 are even better than those from GAFF2/RESP. Figure S5A,B in the Supporting Information show the scatter plots of heats of vaporization calculated by GAFF2/RESP or GAFF2/ABCG2 versus the experimental values. Figure S5C shows the scatter plot of heats of vaporization calculated by GAFF2/RESP versus the values calculated by GAFF2/ABCG2, which demonstrates a very high correlation: MSD = 0.18 kcal/mol, MUD = 0.69 kcal/mol, RMSD = 1.24 kcal/mol, and *R* = 0.951.

Besides the large scale of benchmarking bulk properties, we also randomly selected 96 real drug molecules from the DrugBank database (https://go.drugbank.com/), generated 5 different conformations for each drug molecule using the OpenEye’s Omega2 module (https://docs.eyesopen.com/applications/omega/), and generated atomic charges from different force fields: first, the Charmm general force field (CGenFF) using the online tool Ligand Reader & Modeler on the website of CHARMM-GUI (https://www.charmm-gui.org/?doc=input/ligandrm); second, the OPLS3 force field using Maestro Suite 2017–2 from Schrodinger Inc. (https://www.schrodinger.com/platform/products/maestro/); and third, our GAFF2/ABCG2 combination using the modified Antechamber module in the AmberTools24 (https://ambermd.org/AmberTools.php). Note that the generated partial atomic charges from CGenFF are determined by the atom types and connections, thus independent of the input conformations.^68^ In order to conduct a fair comparison of calculated dipoles and quadrupoles from different molecular mechanics (MM) FFs against those from quantum mechanics (QM) at the HF/6-31G* level, two sets of ABCG2 charges were generated for these drug molecules. In set one (hereafter referred as abcg2_sc), only one single conformation of each drug molecule was adopted to generate atomic charges, and the generated charges were directly copied to other conformations of the same molecule. In set two (hereafter referred to as abcg2), every conformation of a drug molecule was adopted separately to generate atomic charges. The RMSE and the correlation R2 of calculated MM dipoles (dx, dy, dz, dm) and quadrupoles (qxx, qyy, qzz, qxy, qxz, qyz) against the QM values over the different conformations for each of the 96 drug molecules are listed on the sheet of “dipole_drugs” in the spreadsheet file of the Supporting Information, and the statistical results over all 96 molecules are shown at the bottom position of the same sheet. From the statistical results, we can see that the abcg2_sc set achieved most of the smallest RMSE and the highest correlation among the dipoles and quadrupoles (colored cyan) compared to the corresponding results from CGenFF and OPLS3. In general, the results from ABCG2 are better than those from OPLS3 and are much better than those from CGenFF in reproducing HF/6-31G* dipole and quadrupole moments. We can also notice that the RMSEs and correlations from set abcg2 (charges were generated separately for each conformation) are generally further improved against set abcg2_sc (charges were generated from a single conformation and then copied to other conformations).

In the Introduction section, we cited the claim by the authors of AM1-BCC^39,40^ that one advantage of AM1-BCC (and ABCG2) charge methods is that they are less conformationally dependent compared to the methods of fitting electrostatic potentials like the default RESP method for GAFF and GAFF2. This advantage is rooted in the Mulliken populations of electric charge distribution, based on which the AM1-BCC and ABCG2 charge models apply various bond charge corrections (BCCs). Here we provide a typical example to demonstrate the point. Polyalcohol molecules have multiple hydroxyl groups (−OH) on neighboring carbon atoms and can form complicated intramolecular hydrogen bonds (H bonds); therefore, different conformations may have quite different charge distributions in principle. Ethylene glycol (Figure S6) is the smallest form of polyalcohol compounds and has ten unique conformations based on its three consecutive dihedral angles (H–O–C–C, O–C–C–O, and C–C–O–H).^69^ We applied both RESP and ABCG2 charge models to assign partial charges on ethylene glycol (see the spreadsheet file in the Supporting Information) using the ten unique conformations as the input. The standard deviations (STDs), here called as fluctuations, of assigned atomic charges among the different conformations from the RESP method are 0.0246 e for O, 0.0078 e for H in −OH, 0.0219 e for H in −CH_2_–, and 0.0475 e in C; the corresponding STD values from the ABCG2 method are much smaller: 0.0062 e for O, 0.0057 e for H in −OH, 0.0056 e for H in −CH_2_–, and 0.0137 e for C. The averaged value over the total 10 atoms on the fluctuations of assigned atomic charges over unique conformations by the RESP method is 0.0247 (Table S3), and the corresponding averaged fluctuation value from our ABCG2 methods is only 0.0073 (Table S3).

Besides the exhibition of the above typical molecule of ethylene glycol, we also similarly calculated the averaged fluctuations of atomic charges of the aforementioned 96 real drug molecules over 5 different conformations from the RESP and ABCG2 methods (see the sheet of “charge_fluctuation” in the spreadsheet file in the Supporting Information). It is evident that the averaged fluctuation value from the ABCG2 method is smaller than that of the RESP method for every drug molecule, demonstrating that the ABCG2 method is less conformationally dependent than RESP. The mean values of averaged charge fluctuations on all 96 drug molecules are 0.0230 and 0.0045 for RESP and ABCG2 (Table S3), respectively.

We compared the atomic charges for all 642 solute molecules in the FreeSolv database generated by the ABCG2 charge model and the RESP charge model, as shown in Figure S7. The correlation between ABCG2 atomic charges and RESP atomic charges is 0.79 for 5600 heavy atoms, 0.84 for 6013 hydrogen atoms, and 0.83 for all atoms, respectively.

All of the discussed solutes in this study are neutral organic compounds. We understand that there are high needs for similar charge assignment protocols and parameters for charged organic molecules. However, plenty of obstacles exist. The magnitudes of experimental hydration/solvation free energies for charged molecules are usually much higher than those of neutral molecules. The reported experimental data for charged molecules are usually in controversy. There are different discussions and opinions on the correction of the values of experimental measurements and the values from alchemical free energy calculations. As far as we know, there is no reliable curated database of the hydration/solvation free energies of charged organic molecules. As described in our previous article,^13^ we collected experimental hydration free energy data of a few of the simple ammonium and carboxylic ions, developed ABCG2 parameters for them, and listed the results on the sheet of “ions_hydration” in the supplementary spreadsheet file. The corresponding structures are shown in Figure S8. The parametrization for zwitterionic and larger charged molecules is beyond the scope of this manuscript due to the lack of reliable curated databases.

## Conclusions

In summary, we reported our recently developed ABCG2 charge model, which can assign partial atomic charges instantaneously for arbitrary neutral organic molecules, and the assigned charges are less dependent on input conformation than the default RESP method for the second generation of the general AMBER force field (GAFF2). Thermodynamic integration (TI) through molecular dynamics (MD) simulations with the combination of GAFF2/ABCG2 achieved a root-mean-square error (RMSE) of 0.99 kcal/mol on the hydration free energy (Δ*G*_hyd_) calculations of all 642 solutes in the golden-standard FreeSolv v0.52 database. This result broke the threshold of the RMSE of 1 kcal/mol on all solutes in this data set for the first time, as far as we know. When applied on the solvation free energy (Δ*G*_solv_) calculations on diverse organic solutes in various organic solvents (without any special “tweaks” on solvent molecules), GAFF2/ABCG2 obtained an RMSE of 0.89 kcal/mol on the 2068 organic solvent–solute pairs in the Minnesota Solvation database version 2012 (MNSol-v2012), again broke, and went beyond the threshold of an RMSE of 1 kcal/mol on such a large scale of the Δ*G*_solv_ benchmark. The combination of our already popular additive force field GAFF2 with this newly developed semiempirical ABCG2 charge model achieved a better mean unsigned error (MUE) than a polarizable force field (FF) AMOEBA and a slightly worse MUE than another state-of-the-art multipolar polarizable ARROW FF, which is used in ab initio MD simulations including nuclear quantum effects, on a selected small data set of organic molecules with typical functional groups. An additional benchmark on the 685 Δ*G*_hyd_ on the ATB3.0 water validation data set achieved an RMSE of only 0.79 kcal/mol. The derived transfer free energy (Δ*G*_trans_) from the aqueous solution to the organic solvent on 1913 data points obtained an RMSE of 0.85 kcal/mol, corresponding to 0.63 log units for the partition coefficient logP. The breakthrough accuracy and transferability among different polar and nonpolar solvents of GAFF2/ABCG2, plus the convenience and comprehensiveness for possibly encountered chemical functional groups of arbitrary organic molecules, give a great promise on the accurate prediction of other important physicochemical properties related to Δ*G*_solv_, such as the solubility, partition and distribution coefficients, membrane permeability, and protein–ligand binding free energy, which are all greatly useful in the discovery and development of medicines. Besides the large scales of benchmark calculations on Δ*G*_hyd_/Δ*G*_solv_/Δ*G*_trans_, we also carried out large scales of benchmark calculations on properties of pure bulk liquids like densities (1839 data points) and heats of vaporizations (874 data points) and illustrated the reliability of calculating these properties by GAFF2/RESP and GAFF2/ABCG2. The success of this work further strengthens our belief that computationally feasible fixed-charge FFs can be further improved to achieve even higher accuracy. In the near future, we plan to further benchmark the performance of GAFF2/ABCG2 on properties like membrane permeability, ADMET, and protein–ligand binding affinities. We also plan to fully optimize the bonded parameters and nonbonded van der Waals parameters in our current GAFF2 based on this ABCG2 charge model to finish the development of the next generation of the general AMBER force field, GAFF3. GAFF3 is expected to have broad applications in computer-aided drug design (CADD) projects, given its outstanding performance in free energy calculations and on-the-fly atomic charge assignment when in combination with a machine learning trained Milliken population charge model.

## Data Availability

Raw data are included in the EXCEL spreadsheet file in the Supporting Information except for the experimental data of the MNSol 2012 database. Readers should apply for a license from https://comp.chem.umn.edu/mnsol/, which is free to academic groups, in order to get the experimental data of MNSol 2012. Molecular structures of solutes and solvents in the mol2 format, the corresponding topology files (prepi) with assigned ABCG2 charges and parameter files (frcmod), and examples of Amber input files are included in the supplemented tgz file accessible from https://github.com/junmwang/abcg2. Force field and charge assignment parameters are also available through the updated Antechamber module with a new option of “-c abcg2″, which have been released with AmberTools24 in 2024 and which is free to the public (https://ambermd.org/GetAmber.php).

## Supporting Information Available

Performance of GAFF2/RESP and GAFF2/ABCG2 on reproducing densities of 1839 bulk liquids; scatter plots; benchmark of heats of vaporization of 874 pure liquids with GAFF2/RESP andGAFF2/ABCG2; ethylene glycol (PDF)
Related raw data (XLSX)

## Abbreviations

AM1-BCC: Austin Model 1 with bond charge correction
BAR: Bennett acceptance ratio
CGenFF: CHARMM general force field
DFT: density functional theory
FEP: free energy perturbation
FF: force field
GAFF: general AMBER force field
MBAR: multistate Bennett acceptance ratio
MD: molecular dynamics
ML: machine learning
MM: molecular mechanics
MSD: mean signed deviation
MSE: mean signed error
MSPE: mean signed percent error
MUD: mean unsigned deviation
MUE: mean unsigned error
MUPE: mean unsigned percent error
QM: quantum mechanics
RESP: restrained electrostatic potential
RMSE: root-mean-square error
RMSPE: root mean squared percent error
SAMPL: statistical assessment of the modeling of proteins and ligands
TI: thermodynamic integration
