# Supplementary material for: ABCG2: A Milestone Charge Model for Accurate Solvation Free Energy Calculation
Source: J Chem Theory Comput. 2025 Mar 11;21(6):3032–43. doi: 10.1021/acs.jctc.5c00038 (PMC11948320; doi:10.1021/acs.jctc.5c00038)
Supplement: Supplementary file 1 — ct5c00038_si_001.pdf [file ct5c00038_si_001.pdf]

Supporting Information

ABCG2: a Milestone Charge Model for Accurate Solvation Free Energy Calculation.

Xibing He[1], Viet H. Man[1], Wei Yang[2], Tai-Sung Lee[3], Junmei Wang[1,*]

[1]Department of Pharmaceutical Sciences and Computational Chemical Genomics Screening Center, School of Pharmacy, University of Pittsburgh, Pittsburgh, PA 15261, USA.

[2]Department of Chemistry and Biochemistry and Institute of Molecular Biophysics, Florida State University, Tallahassee, Florida 32306, USA.

[3]Laboratory for Biomolecular Simulation Research, Center for Integrative Proteomics Research, and Department of Chemistry and Chemical Biology, Rutgers University, Piscataway, New Jersey 08854, USA.

*Junmei Wang.
Email: Juw79@pitt.edu

This file includes:

Supporting text S1: FreeSolv is a well-established, golden-standard database of hydration free energies.
Tables S1 to S3.
Figures S1 to S8.

Supporting Information Text

Text S1. FreeSolv is a well-established, golden-standard database of hydration free energies.

One major obstacle to hindering the development of force fields (FFs) and charge models is the scarcity of reliable experimental data of hydration free energy (ΔG_{hyd}) and solvation free energy (ΔG_{solv}). Despite the important role of ΔG_{solv} in multiple research fields such as chemical reactions, biophysical processes like protein folding and protein-ligand binding, environmental issues like pollute distribution, etc., the experimental data of ΔG_{solv}, especially ΔG_{hyd}, are very limited and scattered in a variety of journals, and it needs considerable work to gather, compare, and curate into a reliable database. Frequently experiment data on the same pair of solvent and solute from different resources can differ considerably, and discrepancy larger than 1 kcal/mol is not uncommon. In such cases, careful examining of the controversial data by experimental experts and extra experimental verification are critical. Unfortunately, accurate experimental measurements of $\Delta G_{hyd}/\Delta G_{solv}$ are difficult and expensive, but somehow are considered as an outdated field in terms of topic hotness and technique innovation, hence the significance of such work is under-valued by fund providers, leading to less and less devoted scientists in this field. For example, Dr. J. P. Guthrie, as an experimental expert on hydration/solvation, carried out curation work on ΔG_{hyd} and composed a database called FreeSolv (https://github.com/MobleyLab/FreeSolv). Unfortunately, Dr. Guthrie passed away at age 76 in 2017, and left unfinished work with a larger amount of un-curated data (https://github.com/MobleyLab/GuthrieSolv). The FreeSolv database version 0.52 contains experimental ΔG_{hyd} of 642 neutral organic solutes, which is probably the largest reliable experimental database on ΔG_{hyd} which is curated by experimental experts. Hence FreeSolv has been used as a well-established, golden-standard benchmarking dataset by numerous researchers in the field of molecular modelling, molecular machine learning (ML), etc.

Table S1. The performance of GAFF2/RESP and GAFF2/ABCG2 on reproducing densities of 1839 bulk liquids.

Charge method	MSE (g/cm^3)	MUE (g/cm^3)	RMSE (g/cm^3)	MSPE (%)	MUPE (%)	RMSPE (%)
RESP	0.010	0.022	0.036	1.10	2.21	3.54
ABCG2	0.011	0.023	0.037	1.19	2.24	3.56

Table S2. The performance of GAFF2/RESP and GAFF2/ABCG2 on reproducing the heats of vaporization of 874 organic liquids

Charge method	MSE (kcal/mol)	MUE (kcal/mol)	RMSE (kcal/mol)	MSPE (%)	MUPE (%)	RMSPE (%)
RESP	-0.84	1.54	1.88	-7.08	14.08	17.55
ABCG2	-0.67	1.38	1.74	-5.30	12.70	16.20

Table S3. The averged fluctuations of assigned partial charges over all atoms and over different conformations for the ethylene glycol molecules and for 96 real drug molecules.

Charge method	Ethylene glycol	Drug molecules
RESP	0.0247	0.0230
ABCG2	0.0073	0.0045

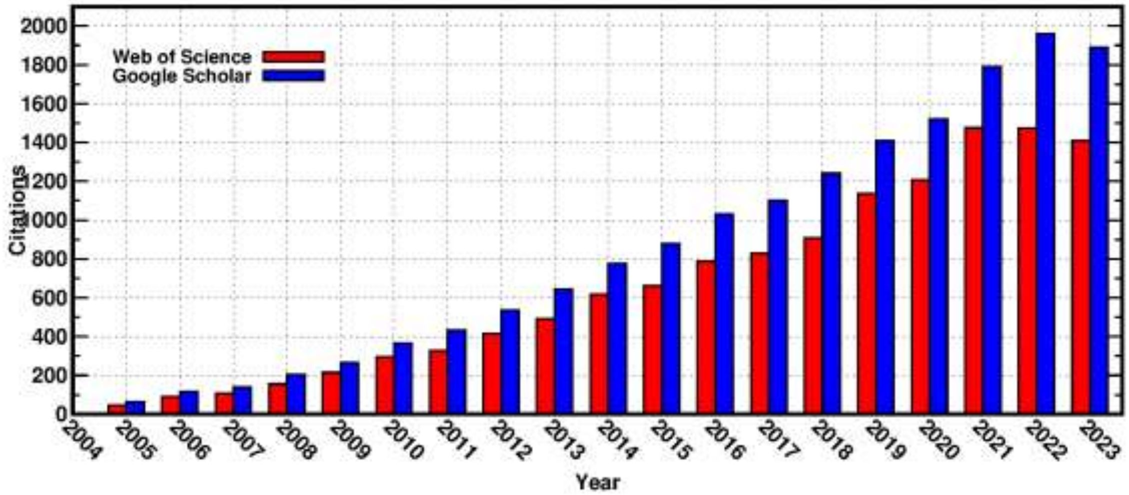

Figure S1. Citations of our GAFF paper (DOI10.1002/jcc.20035) by 01/24/2024. The total citation is 12,637 according to Web of Science, and 16,474 according to Google Scholar. Since we haven't written and published an article specifically about GAFF2 since its release in 2015, the GAFF2 users usually cite this GAFF paper.

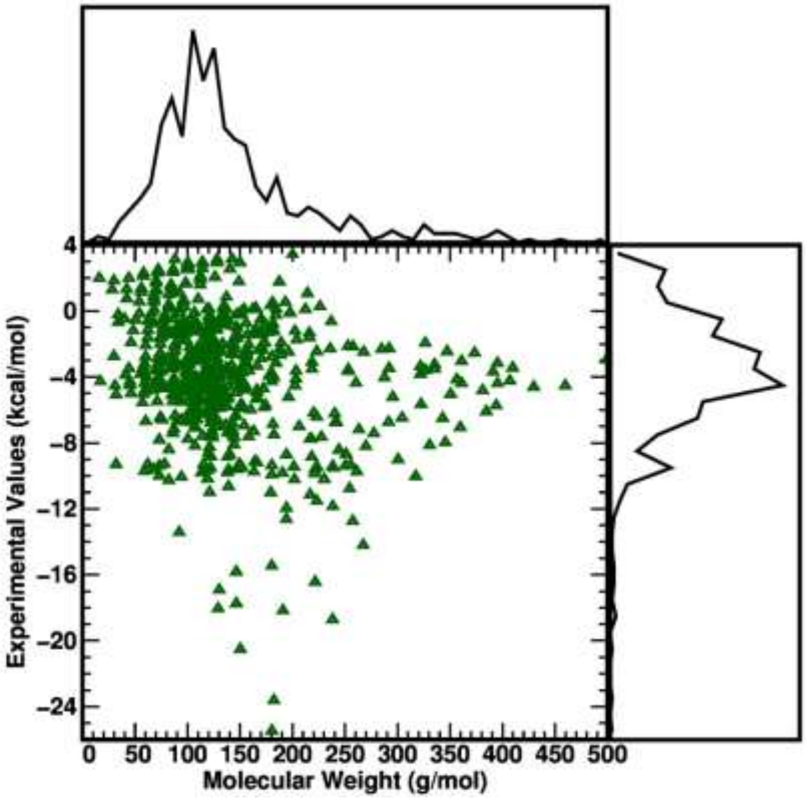

Figure S2. The scatter plot and distribution plots of the molecular weights and experimental hydration free energies of the 642 solutes in the FreeSolv database.

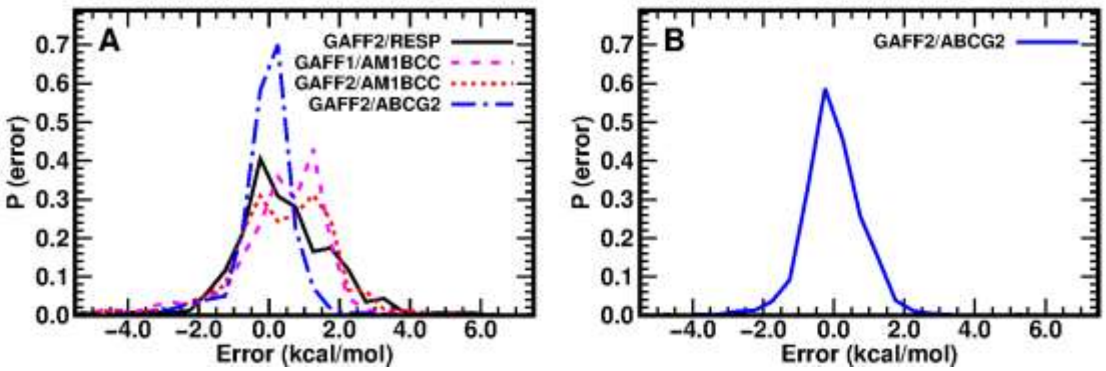

Figure S3. (A) The probability density distribution of signed errors of hydration free energies calculated with various combinations of force fields and charge models on the full FreeSolv data set (642 solutes). (B) The probability density distribution of signed errors of solvation free energies calculated with GAFF2/ABCG2 on on MNSol data set (2068 pairs of solvent-solute).

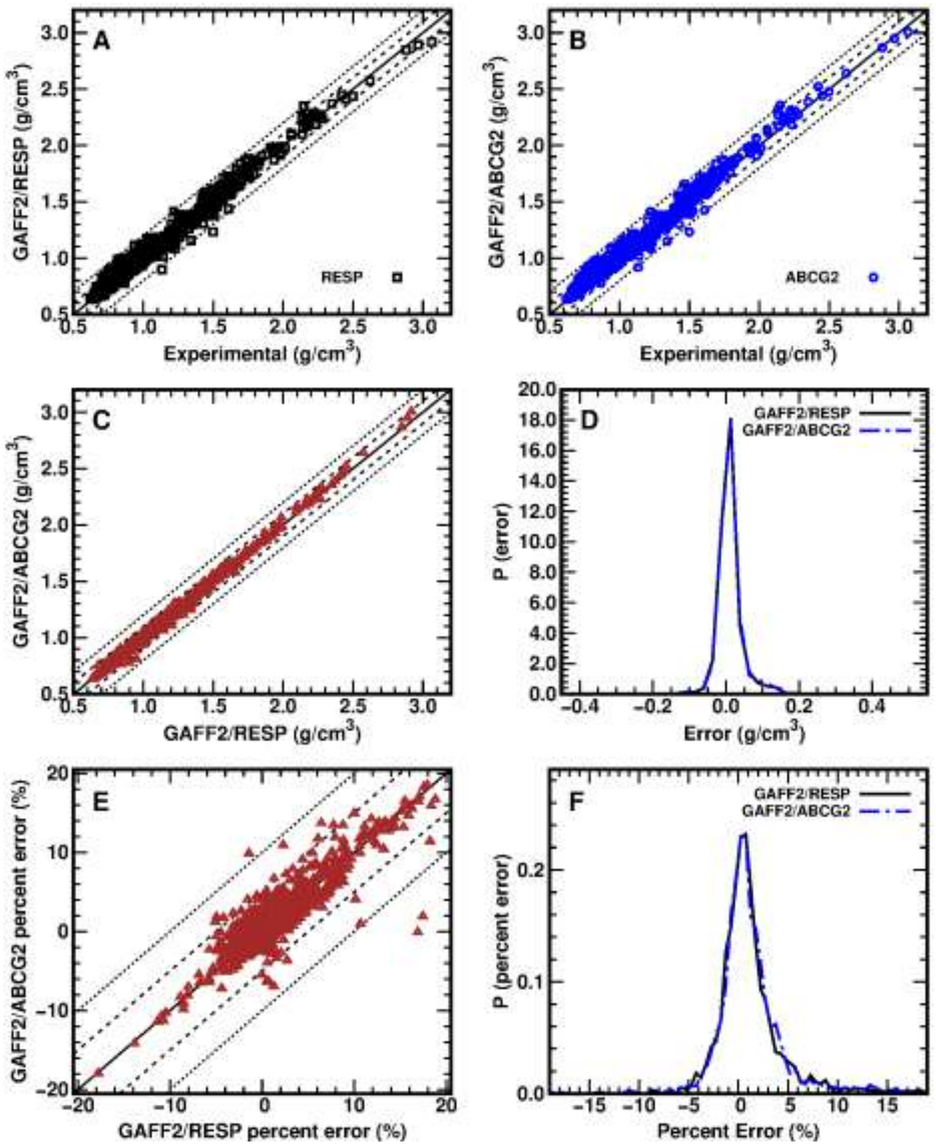

Figure S4. Benchmark of density calculations of 1839 pure liquids with GAFF2/RESP and GAFF2/ABCG2. (A) Densities calculated using GAFF2/RESP versus experimental data. (B) Densities calculated using GAFF2/ABCG2 versus experimental data. (C) Correlations between calculated densities from GAFF2/RESP and GAFF2/ABCG2. (D) The probability density distributions of signed errors. (E) Correlations between percent errors from GAFF2/RESP and GAFF2/ABCG2. (F) The probability density distributions of percent errors.

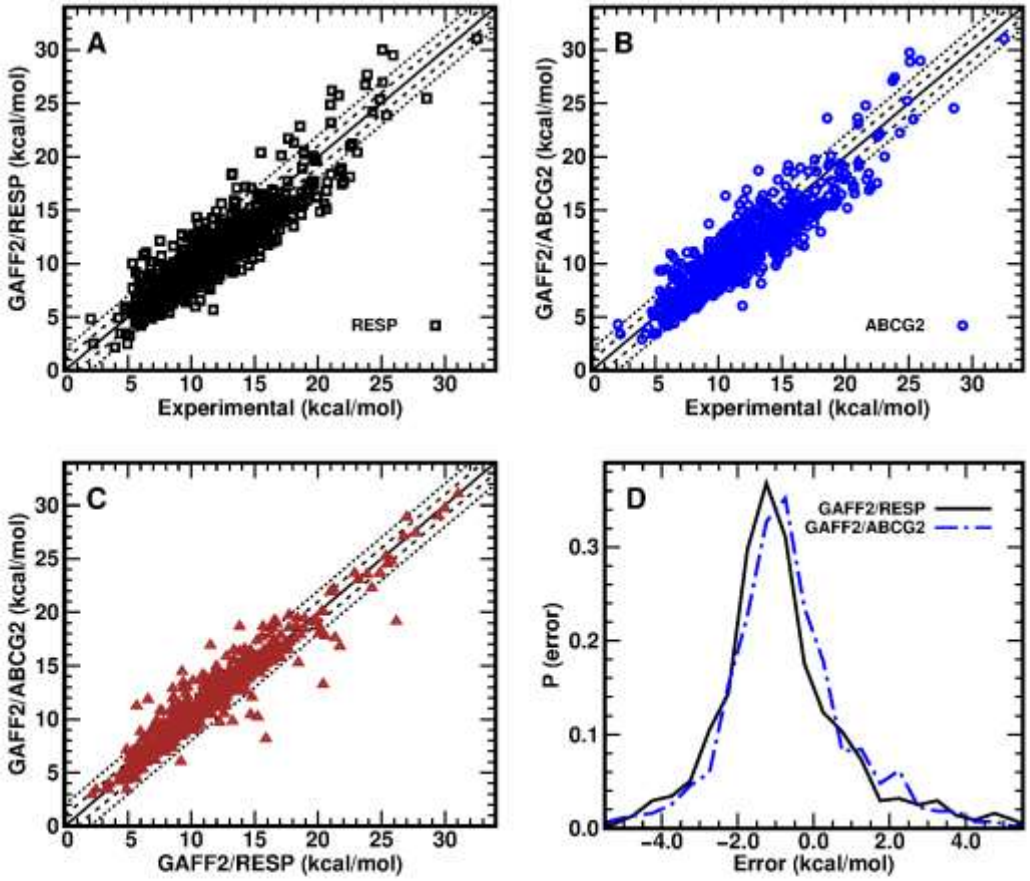

Figure S5. Benchmark of heats of vaporization of 874 pure liquids with GAFF2/RESP and GAFF2/ABCG2. (A) Heats of vaporization calculated using GAFF2/RESP versus experimental data. (B) Heats of vaporization calculated using GAFF2/ABCG2 versus experimental data. (C) Correlations between calculated heats of vaporization from GAFF2/RESP and GAFF2/ABCG2. (D) The probability density distributions of signed errors.

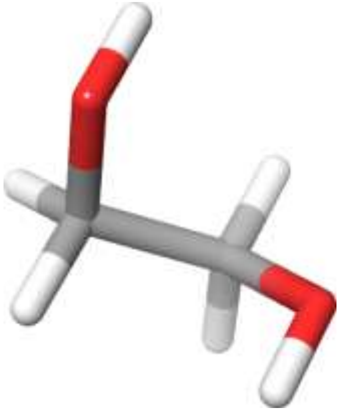

Figure S6. Ethylene glycol.

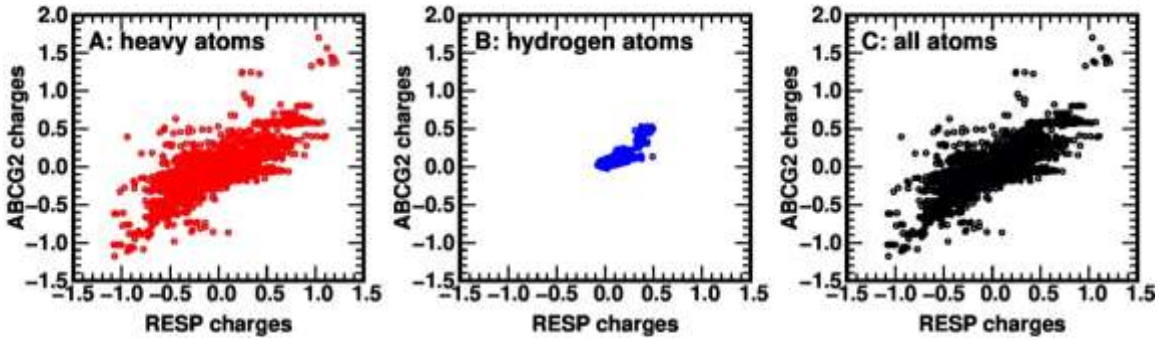

Figure S7. Comparison of ABCG2 atomic charges versus RESP atomic charges for the 642 molecules in the FreeSolv database. A, only heavy atoms, 5600 data points, correlation R = 0.79; B, only hydrogen atoms, 6013 data points, correlation R = 0.84; C, all atoms, 11613 data points, correlation R = 0.83.

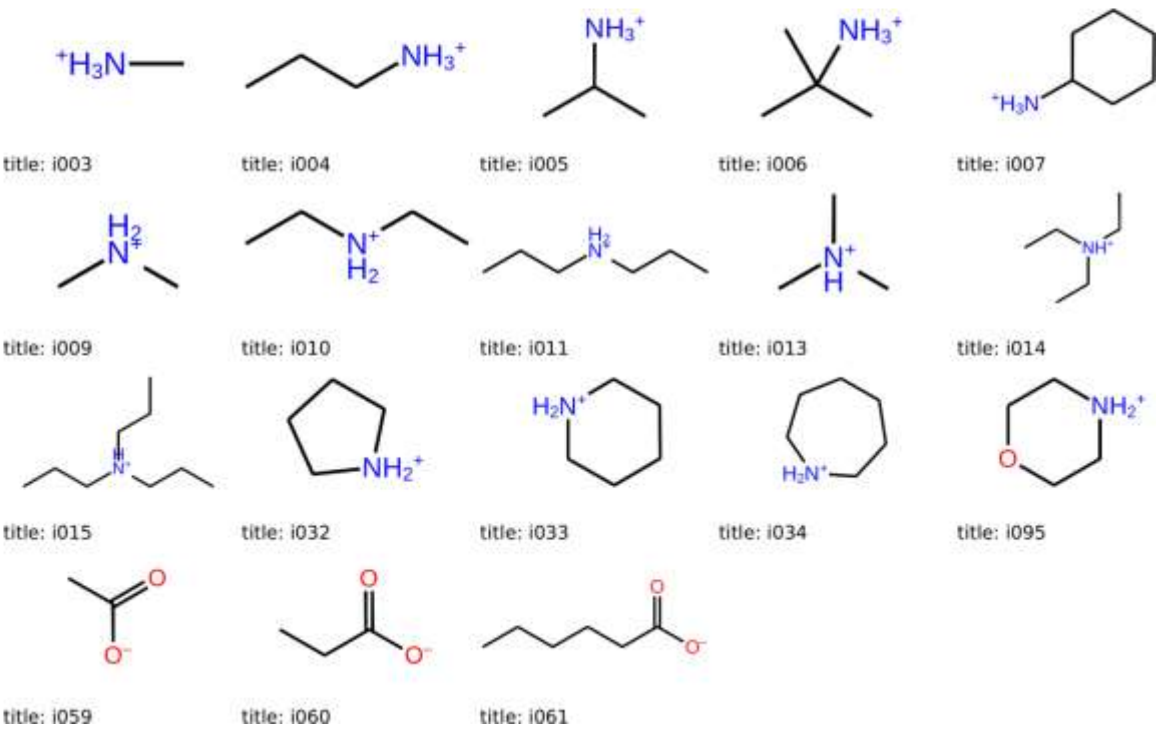

Figure S8. Structures of charged ammoniums and carboxylic acids adopted for hydration free energy calculations.
